# 

**Published:** 1851-10

**Authors:** 


*Medical Department of the University of Buffalo.* — The *preliminary* term in this Institution, commences on the 8th instant, four weeks before the *regular* term, the latter commencing *on the first Wednesday in November.*

